# Negative regulation of ErbB family receptor tyrosine kinases

**DOI:** 10.1038/sj.bjc.6601500

**Published:** 2004-01-20

**Authors:** C Sweeney, K L Carraway

**Affiliations:** 1 UC Davis Cancer Center, University of California, Research Building III, Room 1400, 4645 2nd Avenue, Davis, Sacramento CA 95817, USA

**Keywords:** receptor tyrosine kinase

## Abstract

Receptors of the EGF receptor or ErbB family of growth factor receptor tyrosine kinases are frequently overexpressed in a variety of solid tumours, and the aberrant activation of their tyrosine kinase activities is thought to contribute to tumour growth and progression. Much effort has been put into developing inhibitors of ErbB receptors, and both antibody and small-molecule approaches have exhibited clinical success. Recently, a number of endogenous negative regulatory proteins have been identified that suppress the signalling activity of ErbB receptors in cells. These include intracellular RING finger E3 ubiquitin ligases such as cbl and Nrdp1 that mediate ErbB receptor degradation, and may include a wide variety of secreted and transmembrane proteins that suppress receptor activation by growth factor ligands. It will be of interest to determine the extent to which tumour cells suppress these pathways to promote their progression, and whether restoration of endogenous receptor-negative regulatory pathways may be exploited for therapeutic benefit.

The ErbB family of receptor tyrosine kinases, consisting of the EGF receptor, ErbB2, ErbB3 and ErbB4, directs a broad range of developmental events and contributes to the malignancy of a number of tumour types. Numerous *in vivo* and *in vitro* studies have shown that ErbB signalling plays essential roles in the development and maintenance of mammary, cardiac and neural tissues, and contributes to the development of a variety of other tissues through epithelial remodelling and differentiation. The aberrant activation of ErbB receptors in tumours can occur via mutational activation, receptor overexpression, or through tumour production of autocrine growth factor ligands.

Owing to the widespread role of ErbB receptor activation in tumour growth and progression, the development of ErbB inhibitors has been a subject of intense interest. Indeed, neutralising antibodies directed towards the EGF receptor or ErbB2 are either in clinical trials or have received FDA approval for the treatment of some tumours (Arteaga, 2003). Moreover, a variety of small-molecule inhibitors are being developed to target these receptors. In this light, it is of interest to look more closely at endogenous ErbB receptor-negative regulatory pathways, the mechanisms by which tumours could overcome these checks on receptor activity, and whether these mechanisms might be employed to ultimately benefit patients.

Members of the mammalian ErbB family are acted upon by EGF-like growth factor ligands, which bind to receptor extracellular domains to stimulate tyrosine kinase activity. The EGF-like domain, a 40–60 amino-acid domain characterised by six cysteine residues forming three disulphide bonds, is a very common component of the extracellular regions of a variety of proteins encompassing a wide range of functions. Only a small subset of EGF-like proteins function as activating ligands for ErbB receptors. Despite a high degree of sequence similarity, each of the ErbB receptors appears to possess distinct biochemical and biological properties. Ligand specificity is determined by the receptor extracellular domains, and EGF receptor, ErbB3 and ErbB4 each bind subsets of the known ligands. No diffusible ligand has been demonstrated to interact with ErbB2. On the other hand, although all four receptors undergo ligand-stimulated homo- and heterodimerisation events as part of their signalling mechanism, ErbB2 appears to be the preferred heterodimerisation partner for the other family members. ErbB3 is unique in that it lacks an intrinsic kinase activity and must necessarily heterodimerise with other family members to propagate signals. Pertinent to a discussion of negative regulatory mechanisms, it has been reported that only EGF receptor undergoes significant ligand-stimulated receptor internalisation and degradation (Baulida *et al*, 1996; Baulida and Carpenter, 1997). The marked differences in the properties of the ErbB receptors point to the possibility that inhibitory mechanisms may not adhere to a one-size-fits-all paradigm; cells may employ different inhibitory mechanisms to negatively regulate the different ErbB family members. Our purpose here is to discuss the variety of mechanisms that directly impinge upon ErbB receptors to regulate their potential to contribute to tumorigenic processes.

## RECEPTOR DEGRADATION

One of the primary mechanisms by which cells negatively regulate growth factor receptor activity is through receptor degradation. For a quarter century, the EGF receptor has served as a prominent model for understanding how cell surface receptors undergo ligand-stimulated downregulation and degradation. In general, ligand stimulation results in receptor localisation to plasma membrane clathrin-coated pits, internalisation of coated pits and delivery to endosomes, and sorting of endosomal receptors for trafficking back to the cell surface or to lysosomes for degradation.

Recent years have seen marked advances in our understanding of the molecular mechanisms underlying these events (Shtiegman and Yarden, 2003). A key protein that has received a great deal of attention in this process is cbl, which contains a phosphotyrosine binding domain and a RING finger E3 ubiquitin ligase domain. Overexpression of cbl in cells augments EGF-stimulated receptor ubiquitylation and degradation, and functional RING finger and phosphotyrosine binding domains are both required for enhanced degradation (Levkowitz *et al*, 1998).

Receptor stimulation by EGF results in the phosphorylation of several tyrosine residues of the receptor intracellular region, including tyrosine 1045 that acts as a docking site for cbl. Alternatively, cbl may be recruited in complex with the adaptor protein Grb2 through a distinct phosphotyrosine residue (Waterman *et al*, 2002). Recruited cbl becomes tyrosine phosphorylated by the receptor, activating its ubiquitin ligase activity (Levkowitz *et al*, 1999). cbl is thought to ubiquitylate the receptor on intracellular lysine residues, perhaps through the attachment of multiple monoubiquitin moieties (Mosesson *et al*, 2003), to mediate receptor trafficking. Interestingly, some EGF receptor stimulants such as oxidants that promote robust gross receptor tyrosine phosphorylation do not significantly stimulate the phosphorylation of tyrosine 1045. This leads to enhanced receptor stability, and the inability of the EGF receptor to become downregulated following oxidant exposure could contribute to the progression of diseases such as lung cancer (Ravid *et al*, 2002).

**Figure 1:**
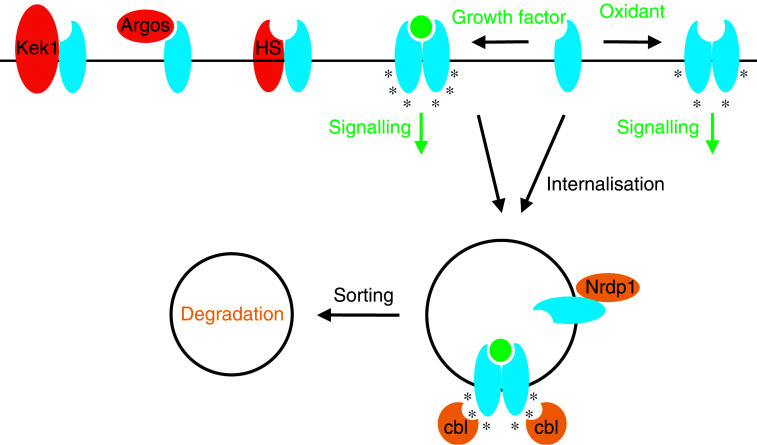
ErbB receptor-negative regulatory mechanisms. Activation of ErbB receptors (blue) by growth factor binding leads to receptor dimerisation and phosphorylation on several tyrosine residues (asterisks), while activation by oxidants leads to a subset of receptor phosphorylation events. Active and inactive receptors are internalised, where they may be acted upon by E3 ubiquitin ligases. Ubiquitylation of unoccupied ErbB3 and ErbB4 after Nrdp1 binding marks these receptors for degradation. Cbl binds to tyrosine 1045 of ligand-activated EGF receptor to facilitate receptor ubiquitylation and trafficking to lysosomes. Tyrosine 1045 of oxidant-activated EGF receptor is not phosphorylated, and these receptors are resistant to downregulation. Kek1, argos and herstatin (HS) are all examples of transmembrane or secreted proteins that interfere with ErbB receptor activation, either by disrupting ligand binding or by interfering with the dimerisation or activation mechanism.

The cellular site(s) of cbl action toward the EGF receptor and its role in internalisation are a point of controversy. While evidence suggests that cbl is capable of interacting with the receptor prior to localisation in coated pits (de Melker *et al*, 2001), and that attachment of a single ubiquitin moiety to the carboxy terminus of the receptor may be sufficient to induce receptor internalisation and degradation (Mosesson *et al*, 2003), studies using cells derived from mice lacking a functional cbl gene suggest that cbl-mediated ubiquitylation is not necessary for EGF-stimulated internalisation (Duan *et al*, 2003). It is generally agreed, however, that cbl-mediated receptor ubiquitylation targets endosomal receptors for degradation in the lysosome, while nonubiquitylated receptors are routed back to the cell surface (Figure 1

).

Interestingly, cbl has been reported to be an inefficient substrate of the other ErbB receptors under physiological conditions (Levkowitz *et al*, 1996). These observations are consistent with observations that ErbB2, ErbB3 and ErbB4 do not undergo significant ligand-induced downregulation, and underscore the importance of other mechanisms in keeping the activities of these receptors in check. In the absence of ligand-induced receptor degradation mechanisms, it becomes much more important to precisely control cell surface receptor levels. This will ensure sufficient signalling for cellular events directed toward tissue development or maintenance, yet prevent oversignalling that could lead to disease. While to a first approximation, cell surface receptor levels can be controlled at the transcriptional level, recent studies suggest that receptor ubiquitylation by novel E3 ubiquitin ligases may also play a role in the maintenance of steady-state receptor levels.

Even in the absence of ligand binding, growth factor receptors undergo constant internalisation and trafficking through endosomes. While most internalised receptors are returned to the cell surface, a small fraction of unoccupied receptors is targeted for degradation. The competing processes of recycling and degradation establish an equilibrium that defines receptor half-life and hence the steady-state levels of cell surface receptors (Wiley, 2003). For example, in a normal epithelial cell, a growth factor receptor may be recycled a dozen times prior to its degradation, resulting in a half-life of 12 h. However, in a transformed cell, a smaller fraction of the trafficked receptors may be targeted for degradation, resulting in a markedly prolonged receptor half-life and elevated cell surface receptor levels. Hence, protein(s) involved in targeting receptors for ligand-independent degradation could play a significant role in suppressing the tumour growth properties by suppressing endogenous receptor levels.

Two recent studies have characterised the role of a novel E3 ubiquitin ligase in controlling the cellular levels of ErbB3 and ErbB4 (Diamonti *et al*, 2002; Qiu and Goldberg, 2002). Nrdp1 was originally identified as a RING finger domain-containing protein that interacts with ErbB3 in a ligand- and phosphotyrosine-independent manner. Coexpression studies demonstrated that Nrdp1 is capable of specifically interacting with ErbB3 and ErbB4, binding receptors for the neuregulin-1 and neuregulin-2 EGF-like growth factors, but not with EGF receptor or ErbB2. Like cbl, Nrdp1 overexpression is capable of specifically suppressing the cellular levels of its target receptors, and both the receptor-binding region and the RING finger domain are necessary for this process. Unlike cbl, Nrdp1 acts similarly on ligand-stimulated and unstimulated receptors, dramatically lowering the half-life of ErbB3. Nrdp1-mediated ubiquitylation of ErbB3 *in vitro* and relocalisation of ErbB3 from the cell surface into intracellular compartments suggest that this protein could act to target the ErbB3 and ErbB4 receptors to degradative compartments, in a manner analogous to cbl-mediated EGF receptor trafficking and degradation.

Physiologically, variations in activities of E3 ligases that mark receptors for degradation could play a role in ensuring that signalling is temporally confined. For example, following a particular growth factor-dependent developmental event, upregulation of an E3 ligase activity that targets the corresponding receptor tyrosine kinase could clear the unoccupied or ligand-bound receptors to prevent further signalling. In tumour cells, the suppression of such E3 ligase activity could permit receptor overexpression and facilitate events associated with tumour growth and progression. Receptor-specific sorting E3 ligase activity may be regulated at the transcriptional level, where the negative regulator could be a transcriptional target of receptor activation. Alternatively, it has been observed that Nrdp1 is extremely labile in many cell types and mediates its own ubiquitylation and degradation through a proteasome-dependent pathway (Diamonti *et al*, 2002; Qiu and Goldberg, 2002), raising the possibility that pathways that specifically stabilise E3 ligase proteins could augment receptor degradation.

## NEGATIVE MODULATOR PROTEINS

A second mechanism by which cells negatively regulate ErbB receptors is through the use of extrinsic modulator proteins that physically interact with receptors to determine their response to ligand binding (Carraway and Sweeney, 2001). Modulators can either potentiate receptor response to ligand, illustrated by the augmented signalling observed in the ErbB2–ErbB3 heterodimer in the presence of ASGP2 or CD44 (Carraway *et al*, 1999; Sherman *et al*, 2000), or they can negatively regulate receptor activity.

One class of naturally occurring negative modulators consists of splice variants encoding some or most of the extracellular portions of ErbB receptors. Herstatin is the product of an alternatively spliced human ErbB2 that leaves intron 8 in the message. The resulting expressed product encompasses half of the ErbB2 extracellular region along with 79 amino acids encoded by the retained intron (Doherty *et al*, 1999). Herstatin binds with high affinity to EGF receptor and ErbB2, and inhibits ErbB receptor homo- and heterodimer formation and tyrosine phosphorylation (Azios *et al*, 2001). Interestingly, rather than completely inhibiting ErbB receptor signalling, Herstatin appears to modulate signalling pathway usage by receptors to elicit cellular growth arrest (Justman and Clinton, 2002). A similar splice variant of ErbB3 encoding most of the extracellular region followed by intron-encoded sequence may act as an inhibitor of cell growth by binding ligand to form nonproductive ligand–receptor complexes (Lee *et al*, 2001).

On a more speculative note, studies with the *Drosophila melanogaster* EGF receptor point to the possibility that other negative modulators may exist in humans. Flies have a single EGF receptor family member that can be stimulated by four EGF-like growth factors. In addition, two feedback negative regulators that act directly and specifically on the fly receptor have been identified. The first, called Argos, contains an unconventional EGF-like domain that disrupts the spacing between cysteine residues found in the activating EGF-like ligands. Argos acts as an antagonist of fly EGF receptor activity (Jin *et al*, 2000; Vinos and Freeman, 2000), binding to the receptor with modest affinity and inhibiting activation by other ligands. Argos is expressed in response to EGF receptor activation, and is thought to contribute to receptor inhibition events critical for proper development (Stevens, 1998). Interestingly, it has been reported that potato carboxypeptidase inhibitor (CPI), a 39 amino-acid-secreted protein containing a disulphide-bonded T-knot structure moderately reminiscent of EGF-like domains, acts as an antagonist of the EGF receptor in human tumour cells (Blanco-Aparicio *et al*, 1998). Taken together, these observations point to the possibility that human proteins containing EGF-like domains with unusually spaced cysteine residues, or containing other disulphide-bonded structures, could act as endogenous inhibitory factors for mammalian ErbB receptor family members. Since dozens of proteins encompassing hundreds of EGF-like or cysteine knot domains are encoded by human genes, the list of candidates is quite extensive. Given that some of the activating ligands contain other domains as well, it is possible that ErbB antagonistic domains could be components of proteins typically associated with unrelated functions.

CPI was shown to inhibit EGF binding to the EGF receptor, EGF-stimulated receptor tyrosine phosphorylation and cellular proliferation, and was also demonstrated to inhibit the growth of pancreatic tumour cells in culture and in nude mice. These observations underscore the possibility that, once identified, endogenous ErbB antagonists might ultimately be exploited to inhibit tumour progression in patients.

A second EGF receptor feedback negative regulator identified in *Drosophila* is the leucine-rich repeat (LRR) protein kekkon-1 (Kek1). Kek1 was identified in a screen for downstream targets of fly EGF receptor activation (Musacchio and Perrimon, 1996), and was later found to inhibit EGF receptor signalling *in vivo* and *in vitro* (Ghiglione *et al*, 1999). It is a single-pass transmembrane protein, with a large extracellular region containing an immunoglobulin-like (Ig) domain and a series of six contiguous leucine-rich repeats. Although Kek1 has no EGF-like domain, it physically associates with the fly EGF receptor *in vivo* to suppress receptor signalling (Ghiglione *et al*, 2003). The leucine-rich repeat region of Kek1 is necessary for EGF receptor binding and inhibition. Interestingly, the fly Kek1 protein is capable of interacting with all four members of the mammalian ErbB receptor family, and inhibits signalling through the human EGF receptor (Ghiglione *et al*, 2003). Moreover, it potently inhibits the growth of human breast tumour cell lines and some mouse mammary tumour cell lines in soft agar assays and in xenografts. Specifically, the growth of cell lines derived from mouse tumours arising from activated ErbB2 or from ErbB3 ligand overexpression was inhibited by Kek1 expression, while the growth of a cell line derived from a tumour arising from activated ras was not inhibited.

These results also beg the question of whether or not a human Kek1 homologue exists. Owing to its ability to inhibit ErbB activity, such a protein could be the product of a tumour-suppressor gene whose function is lost in some tumours. The search for a mammalian Kek1 homologue focuses on proteins containing leucine-rich repeats in their extracellular regions. This encompasses a wide variety of proteins such as growth factor receptors, growth factor-binding proteins and proteoglycans, among others. A subset of roughly a dozen of these contains both an LRR region and one or more Ig domains. Given that the Ig domain of Kek1 is dispensable for both EGF receptor binding and suppression of signalling in flies (Ghiglione *et al*, 2003), it is unclear whether the human form would possess this domain. Nonetheless, an attractive candidate called Lig-1 emerges from this list.

Lig-1 was originally identified as the product of a gene whose expression is induced upon retinoic acid-induced differentiation of P19 neuronal embryonic carcinoma cells (Suzuki *et al*, 1996). Its extracellular region contains 15 leucine-rich repeats and three Ig domains. Targeted disruption of the Lig-1 gene in mice yields hyperplastic epidermal lesions reminiscent of psoriasis as a result of keratinocyte hyperproliferation (Suzuki *et al*, 2002), and a comparison of human tissue samples showed a loss of Lig-1 expression in psoriatic skin. Since autocrine activation of the EGF receptor is a major contributor to keratinocyte proliferation in psoriasis, these observations point to the possibility that Lig-1 negatively regulates EGF receptor. Moreover, real-time RT–PCR analysis showed a loss of Lig-1 expression in tumour cell lines relative to normal tissues, raising the possibility that Lig-1 is the product of a tumour-suppressor gene (Hedman *et al*, 2002). However, the impact of Lig-1 on ErbB receptor signalling and on the regulation of tumour cell growth remains to be determined.

Finally, recent evidence suggests that a putative L1-type cell adhesion molecule may interact directly with the *Drosophila* EGF receptor to inhibit its activity. Echinoid, a *Drosophila* transmembrane protein that contains seven Ig domains and two fibronectin type III domains in its extracellular region, was originally identified as a negative regulator of EGF receptor-mediated eye development in flies (Bai *et al*, 2001). This study suggested that Echinoid acts in a separate parallel pathway to negatively regulate signalling downstream of the EGF receptor. However, more recent studies suggest that Echinoid acts at the receptor in flies (Rawlins *et al*, 2003), and that EGF receptor physically associates with Echinoid and mediates its tyrosine phosphorylation when the two proteins are coexpressed in cultured cells (Spencer and Cagan, 2003). In contrast with Kek1 and Argos, Echinoid does not appear to be a transcriptional target of EGF receptor, and it may act through a mechanism that involves inhibition of receptor signalling rather than receptor activation. Despite the actual mechanism, these observations again raise the possibility that a human homologue of Echinoid may be involved in the negative regulation of human ErbB family receptors.

## CONCLUSIONS

It is becoming increasingly clear that cells employ a variety of mechanisms to prevent oversignalling by the ErbB growth factor receptors. Degradation of receptors, mediated by E3 ubiquitin ligases associated with intracellular sorting compartments, may play a central role in suppressing signalling following ligand activation or in maintaining a narrow window of receptors at the cell surface, which are available for ligand activation. Transmembrane and secreted modulator proteins can physically interact with receptors to antagonise ligand binding or to interfere with the receptor activation mechanism. Studies from *Drosophila* offer novel candidates for negative regulators, and newly developed rapid protein–protein interaction screening techniques (Stagljar and Fields, 2002) could facilitate the identification of functional mammalian homologues. In addition, a number of intracellular proteins such as sprouty, Dok, and some tyrosine phosphatases have been shown to suppress signalling following receptor activation. However, such proteins may function more in fine-tuning receptor signalling than in controlling the overall receptor output. It is also apparent that the different ErbB receptor family members are regulated by different mechanisms. For example, the absence of ligand-stimulated neuregulin receptor degradation highlights the need for other negative regulatory mechanisms for these receptors, such as Herstatin and Nrdp1.

While preliminary work has been carried out on some of these negative regulators, the extent to which most of these pathways are suppressed in tumours remains to be explored. It is likely that the function of pathway components, such as E3 ligases associated with receptor expression levels, is lost in tumours associated with ErbB overexpression. The restoration of these pathways, particularly those involving proteins with extracellular regions that interact with ErbB receptors, could offer a novel approach to suppressing the growth properties of ErbB-dependent tumours.
